# Triglyceride-Glucose Index and 30-day mortality in pediatric sepsis: a retrospective cohort study based on PIC database

**DOI:** 10.3389/fped.2026.1704208

**Published:** 2026-02-27

**Authors:** Yi Ding, Tao Mei

**Affiliations:** 1 Graduate School, Jilin University, Changchun, Jilin, China; 2 Department of Pediatrics, Changzhou Maternal and Child Health Care Hospital, Changzhou Medical Center, Nanjing Medical University, Changzhou, Jiangsu, China

**Keywords:** PICU, mortality, pediatric sepsis, PIC database, TyG index

## Abstract

**Background:**

The triglyceride–glucose (TyG) index is widely recognized as a surrogate marker of insulin resistance and poor prognosis in adults. However, the relationship between the TyG index and outcomes in pediatric sepsis patients remains inadequately characterized. Elucidating this association could illuminate the metabolic dimension of sepsis pathophysiology and provide a simple, cost-effective tool for risk stratification in this vulnerable population. This study aims to investigate the relationship between the TyG index and 30-day mortality in pediatric sepsis and to explore its underlying biological significance.

**Methods:**

We conducted a retrospective cohort study and enrolled 149 children who met the diagnostic criteria for sepsis from the PIC database of the Children's Hospital of Zhejiang University between 2010 and 2018. Participants were stratified by TyG level. The primary outcome was 30-day in-hospital all-cause mortality, and the secondary outcome was 30-day ICU all-cause mortality. Cox regression, restricted cubic splines (RCS), and Kaplan–Meier analyses were used to evaluate the association between the TyG index and 30-day mortality in pediatric sepsis patients.

**Results:**

Among the 149 children with sepsis, higher TyG index levels were associated with a reduced 30-day mortality rate. In the multivariate Cox regression model, after adjusting for age, gender and key laboratory variables, the TyG index remained independently and negatively correlated with both in-hospital mortality and intensive care unit mortality. Restrictive cubic spline analysis revealed a linear negative correlation between the TyG index and the risk of death. Subgroup analysis indicated that the TyG index had a consistent protective effect across different age groups, genders and treatment subtypes. Although the Kaplan–Meier survival curve observed a trend of higher TyG index being associated with better survival rates, this association did not reach statistical significance in the sample of this study.

**Conclusions:**

In pediatric patients with sepsis, a higher TyG index was associated with a lower 30-day mortality rate. This finding suggests that the TyG index shows potential for being related to short-term survival rates in children. Future studies need to further explore the interaction between the TyG index and other potential prognostic factors, and verify its value in larger or more diverse populations.

## 1 Introduction

Sepsis, a life-threatening organ dysfunction caused by a dysregulated host response to infection, remains a leading cause of mortality and morbidity in pediatric intensive care units (PICUs) worldwide (1–4). Despite significant advancements in critical care medicine, the mortality rate among pediatric sepsis patients remains alarmingly high, with a substantial proportion succumbing to the disease within the first 30 days of admission. Identifying reliable prognostic markers that can accurately predict the clinical outcomes of pediatric sepsis patients is of paramount importance for early intervention, risk stratification, and optimization of therapeutic strategies.

In recent years, the triglyceride-glucose (TyG) index has emerged as a novel and promising biomarker for assessing metabolic health and predicting adverse outcomes in various clinical settings. The TyG index is calculated using fasting triglyceride and glucose levels, reflecting the combined effects of insulin resistance and dyslipidemia, both of which are core components of metabolic syndrome and are often associated with the pathogenesis of chronic diseases such as type 2 diabetes, cardiovascular disease, and non-alcoholic fatty liver disease (5–8). Increasing evidence suggests that elevated TyG index values are associated with increased risk of cardiovascular events, higher all-cause mortality, and poor outcomes in critically ill adult patients (9–11). However, the relationship between the TyG index and clinical outcomes remains largely unexplored in pediatric sepsis patients admitted to the intensive care unit (ICU).

Given the complex interactions between inflammation, metabolism, and organ dysfunction in sepsis, the TyG index may serve as a valuable prognostic indicator for pediatric sepsis patients. This index may reflect underlying metabolic disorders and systemic stress associated with sepsis, thereby providing insights into disease severity and the risk of poor outcomes. Therefore, this study aims to investigate the association between the TyG index and 30-day mortality in pediatric sepsis patients admitted to the intensive care unit (ICU). By elucidating this relationship, we hope to contribute to the development of more accurate prognostic models in the future and improve the clinical management of pediatric sepsis patients.

## 2 Materials and methods

### 2.1 Study design and setting

This study employed a retrospective cohort design to investigate the association between the triglyceride–glucose (TyG) index and 30-day mortality in pediatric patients with sepsis. Data were obtained from the PIC database, a comprehensive clinical repository of children admitted to the ICUs of the Children's Hospital, Zhejiang University School of Medicine. The database covers a diverse population of pediatric sepsis cases, ensuring the reliability and generalizability of the findings (12). The study was approved by the hospital's Ethics Committee (approval No. 2023-L-043), and the requirement for informed consent was waived because of the retrospective nature of the study and the de-identification of all personal information. In consideration of the compatibility and continuity with the historical data in the database, this study adopted the previously widely accepted diagnostic criteria for sepsis, rather than the latest Phoenix Sepsis Criteria (13). Sepsis was diagnosed based on ICD-10 code.

### 2.2 Data source and patient selection

**Figure 1:**
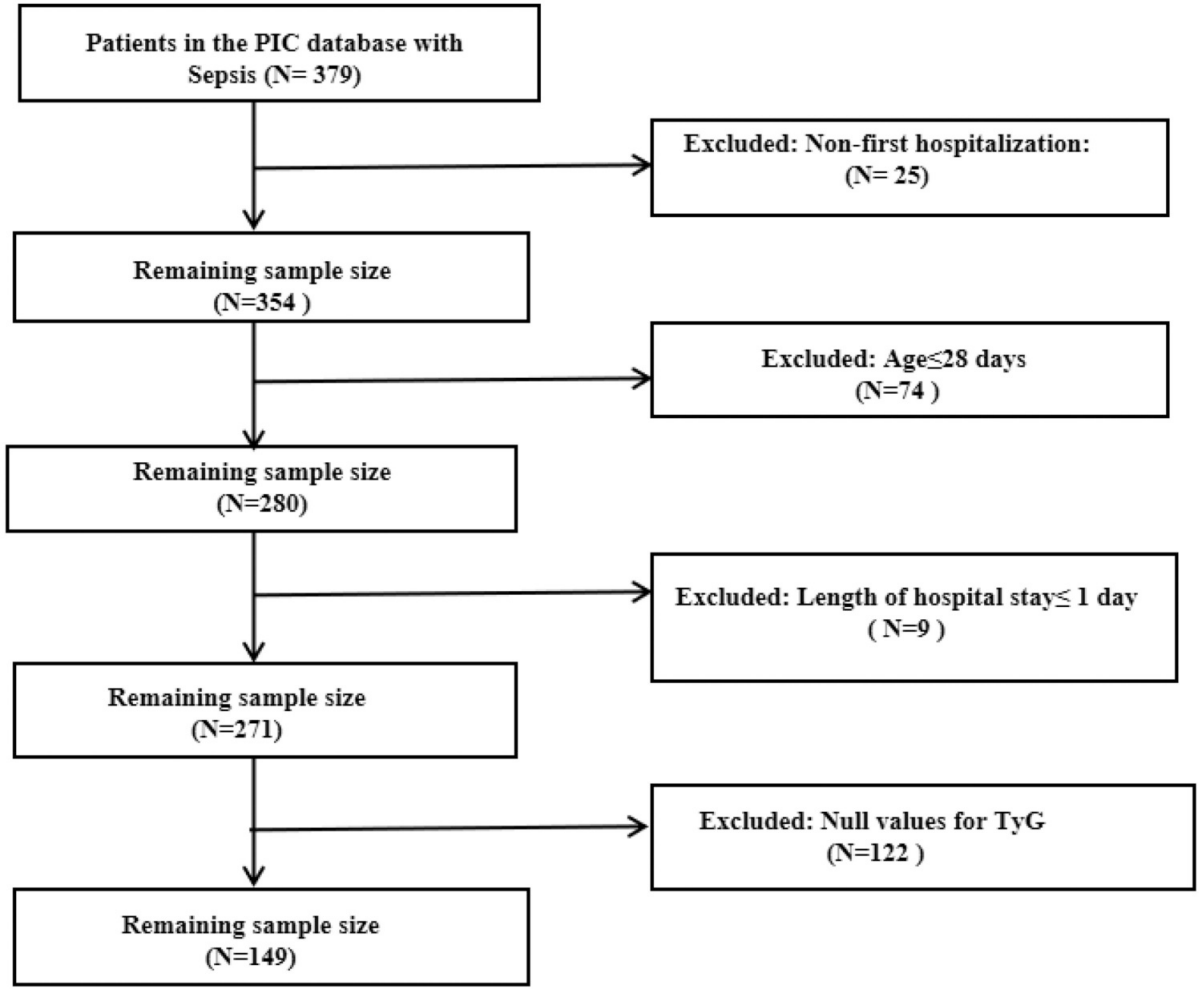
Flowchart of study population selection.

Study participants were drawn from the Pediatric Intensive Care (PIC) database, an open retrospective electronic medical record system developed by the Children's Hospital, Zhejiang University School of Medicine (ZUCH). Detailed information about the database is available on the official website (http://pic.nbscn.org) and in previous publications (12). A total of 149 paediatric patients who met the Sepsis diagnostic criteria were included in the study. The exclusion criteria were as follows: (1) For patients with multiple admissions due to sepsis, only data from the first admission were extracted, excluding 25 patients who were not admitted for the first time (*n* = 25), (2) Patients aged ≤28 days (*n* = 74), (3) Patients with a length of stay in the paediatric intensive care unit of less than 24 h (*n* = 9), (4) Patients with insufficient data (e.g., missing triglyceride and fasting glucose data required for the calculation of the TyG index) (*n* = 122). Ultimately, 149 paediatric patients with sepsis were included in the final analysis. The study population flowchart is presented in Figure 1.

Patients were stratified into two groups (Q1 and Q2) based on the TyG index using the median value as the cutoff point. Specifically, a cutoff value of 8.4 was used to divide the patients into two groups: Q1 (TyG index ≤8.4) and Q2 (TyG index >8.4). This cutoff value was chosen based on the distribution of the TyG index in our dataset and the range commonly used in previous related studies (14). This method is widely accepted due to the lack of a universally recognized standard for the TyG index and allows us to effectively differentiate between high and low TyG index levels in our patient population.

### 2.3 Data collection

The data for this study were directly obtained from the intensive care unit information system, electronic medical records, laboratory results, and vital signs monitors. Patient information from the first day of admission was extracted from the PIC database using Structured Query Language (SQL), including gender, age, comorbidities [such as coronary heart disease (CHD), pneumonia, malignant tumors, and shock], and initial laboratory test results at admission (white blood cell count, creatinine, blood urea nitrogen, cystatin C, serum total cholesterol, C-reactive protein, white blood cell count, neutrophil count, lymphocyte count, hemoglobin, and platelet count). The TyG index was calculated using the formula ln[fasting triglycerides (mg/dl) × fasting blood glucose (mg/dL)/2] (15–18). The measurement of fasting triglycerides and glucose levels was conducted as part of the routine admission procedures. Specifically, blood samples were collected within the first 24 h of admission to the ICU. Although ensuring a strict fasting state in critically ill children is challenging, efforts were made to obtain samples before any significant nutritional intake or during periods when the patients were in a relatively fasting state (e.g., overnight fasting or before morning procedures). The exact timing of sample collection was recorded in the medical records and was within 24 h of admission for all included patients.

### 2.4 Outcome indicators

The primary endpoint of this study was all-cause mortality within 30 days of hospitalization. Secondary endpoints included all-cause mortality within 30 days of ICU admission, defined as death occurring during the ICU stay. For the primary outcome measure, patients were followed up until death, 30 days after admission, or discharge, whichever occurred first. For the secondary endpoints, patients were followed up until death, 30 days after admission, or discharge from the ICU, whichever occurred first.

### 2.5 Statistical methods

This study employed a comprehensive set of statistical methods to elucidate the relationship between the triglyceride-glucose (TyG) index and 30-day mortality in pediatric sepsis patients admitted to the intensive care unit (ICU). Statistical analysis was performed using R version 4.4.1. Descriptive statistics were used to characterize the study population. The Cox proportional hazards model was employed to estimate the hazard ratio (HR) and 95% confidence interval (CI) for the association between the TyG index and 30-day mortality. Additionally, restricted cubic splines (RCS) were used to explore potential non-linear relationships between the TyG index and 30-day mortality. Overall and non-linear *P*-values were reported to determine the significance of the relationship and its linearity. A *p*-value less than 0.05 was considered statistically significant. Kaplan–Meier curves were used to depict survival differences, with a two-sided *P*-value less than 0.05 indicating a statistically significant difference.

## 3 Results

### 3.1 Baseline characteristics and laboratory values

A total of 149 pediatric patients admitted to the intensive care unit (ICU) with sepsis were enrolled and divided into two groups based on the distribution of the TyG index: Q1 (*n* = 73) and Q2 (*n* = 76) (Table 1). Mean total cholesterol was 85.44 mg/dL in Q1 and 109.29 mg/dL in Q2, a statistically significant difference (*p* < 0.001). Except for total cholesterol and the TyG index, baseline characteristics and laboratory parameters were evenly distributed, providing a solid foundation for subsequent analyses of the relationship between the TyG index and ICU mortality in pediatric sepsis.

**Table 1:** Baseline characteristics of the study population.

Variable	Total (*n* = 149)	Q1 (*n* = 73)	Q2 (*n* = 76)	Statistic	*P*-value
TyG	8.24 ± 1.08	7.47 ± 0.98	8.97 ± 0.52	t = −11.64	<0.0001
Sex				*χ*^2^ = 0.00	1.00
Female	60 (40.27)	29 (39.73)	31 (40.79)		
Male	89 (59.73)	44 (60.27)	45 (59.21)		
Age (month)	33.28 ± 43.35	32.68 ± 46.44	33.85 ± 40.45	t = −0.16	0.87
Age category				χ^2^ = 0.45	0.80
1 month-1 year	79 (53.02)	40 (54.79)	39 (51.32)		
1 year-5 years	38 (25.50)	19 (26.03)	19 (25.00)		
over 5 years	32 (21.48)	14 (19.18)	18 (23.68)		
LOS ICU	11.19 ± 14.02	11.74 ± 15.81	10.65 ± 12.15	t = 0.47	0.64
LOS hospital	16.24 ± 15.10	15.84 ± 17.05	16.62 ± 13.05	t = -0.31	0.76
Alb	31.58 ± 7.61	31.48 ± 8.04	31.69 ± 7.22	t = −0.17	0.87
Scr	56.63 ± 56.79	55.71 ± 41.77	57.52 ± 68.46	t = −0.20	0.85
BUN	5.32 ± 3.81	5.17 ± 3.56	5.46 ± 4.05	t = −0.46	0.64
Albumin use				χ^2^ = 0.13	0.72
No	95 (63.76)	45 (61.64)	50 (65.79)		
Yes	54 (36.24)	28 (38.36)	26 (34.21)		
Antibiotic				χ^2^ = 0.00	1.00
No	57 (38.26)	28 (38.36)	29 (38.16)		
Yes	92 (61.74)	45 (61.64)	47 (61.84)		
Cystatin	1.25 ± 0.81	1.14 ± 0.56	1.37 ± 0.99	t = −1.78	0.08
TC	97.60 ± 44.65	85.44 ± 45.51	109.29 ± 40.78	t = −3.36	<0.001
CRP	66.48 ± 64.35	71.63 ± 61.68	61.53 ± 66.85	t = 0.96	0.34
WBC, cell/mm^3^	10.48 ± 11.06	10.25 ± 13.26	10.70 ± 8.52	t = −0.24	0.81
Neutrophil	57.33 ± 24.18	55.82 ± 25.06	58.78 ± 23.38	t = −0.74	0.46
Lymphocyte	27.33 ± 19.19	28.99 ± 21.05	25.74 ± 17.21	t = 1.03	0.31
Hb	82.60 ± 24.11	83.38 ± 26.34	81.86 ± 21.91	t = 0.38	0.70
Plt	184.40 ± 166.00	162.27 ± 133.26	205.66 ± 190.78	t = −1.61	0.11
CHD				χ^2^ = 0.00	0.95
No	140 (93.96)	68 (93.15)	72 (94.74)		
Yes	9 (6.04)	5 (6.85)	4 (5.26)		
Pneu				χ^2^ = 0.04	0.84
No	135 (90.60)	67 (91.78)	68 (89.47)		
Yes	14 (9.40)	6 (8.22)	8 (10.53)		
Cancer				χ^2^ = 0.30	0.58
No	145 (97.32)	70 (95.89)	75 (98.68)		
Yes	4 (2.68)	3 (4.11)	1 (1.32)		
Shock				χ^2^ = 0.15	0.69
No	139 (93.29)	67 (91.78)	72 (94.74)		
Yes	10 (6.71)	6 (8.22)	4 (5.26)		

Q1 and Q2 denote the lower and upper halves of the TyG distribution, respectively. LOS ICU, length of stay in the intensive care unit, LOS hospital, total length of hospital stay, Alb, serum albumin (g L^−^^1^), Scr, serum creatinine (µmol L^−^^1^), BUN, blood urea nitrogen (mmol L^−^^1^), TC, total cholesterol (mg dL^−^^1^), CRP, C-reactive protein (mg L^−^^1^), WBC, white blood cell count (cells mm^−^^3^), Hb, haemoglobin (g L^−^^1^), Plt, platelet count (10^3^ µL^−^^1^), CHD, congenital heart disease, Pneu, pneumonia.

### 3.2 Association of TyG index with mortality in hospitalized and ICU patients

As shown in Table 2, we constructed a Cox proportional hazards model to assess the association between the TyG index and mortality. We then used nested models to progressively adjust for confounding factors. The initial model considered the effect of the TyG index. Model 1 further considered the effects of age and gender on top of the initial model. After adjustment, the 95% confidence intervals of the TyG index were (0.40, 0.72) and (0.40, 0.73), and the *p*-value was still less than 0.0001, indicating that even after considering the effects of age and gender, the association between the TyG index and the mortality rate in the intensive care unit remained significant. Based on the comprehensive consideration of existing literature and biological rationality (14), Model 2 (Model 2) further adjusted for additional laboratory indicators, including white blood cell count (WBC), albumin (Alb), serum creatinine (Scr), blood urea nitrogen (BUN), and total cholesterol (TC), based on Model 1.After adjustment, the 95% confidence interval of the TyG index was (0.43, 0.75), and the *p*-value was still less than 0.0001, indicating that even when these laboratory indicators were taken into account, the association between the TyG index and the mortality rate in the intensive care unit remained significant. From the initial model to Model 2, the 95% CI range of the TyG index remained stable and consistently excluded 1 (i.e., the threshold for no association), further confirming the stability and reliability of the association between the TyG index and ICU mortality.

**Table 2:** Impact of TyG Index on ICU mortality in pediatric sepsis: multivariate models.

Outcome	Crude model		Model 1		Model 2	
	95%CI	*P*	95%CI	*P*	95%CI	*P*
30-day in-hospital mortality (TyG)	0.55 (0.42,0.72)	<0.0001	0.54 (0.40,0.72)	<0.0001	0.57 (0.43,0.75)	<0.0001
30-day in-ICU mortality (TyG)	0.56 (0.43,0.73)	<0.0001	0.54 (0.40,0.73)	<0.0001	0.57 (0.43,0.75)	<0.0001

HR, hazard ratio; CI, confidence interval.

Model 1: adjusted for age (months) and sex.

Model 2: further adjusted for WBC (10^3^/µL), serum albumin (g/L), serum creatinine (µmol/L), BUN (mmol/L) and total cholesterol (mg/dL).

**Figure 2:**
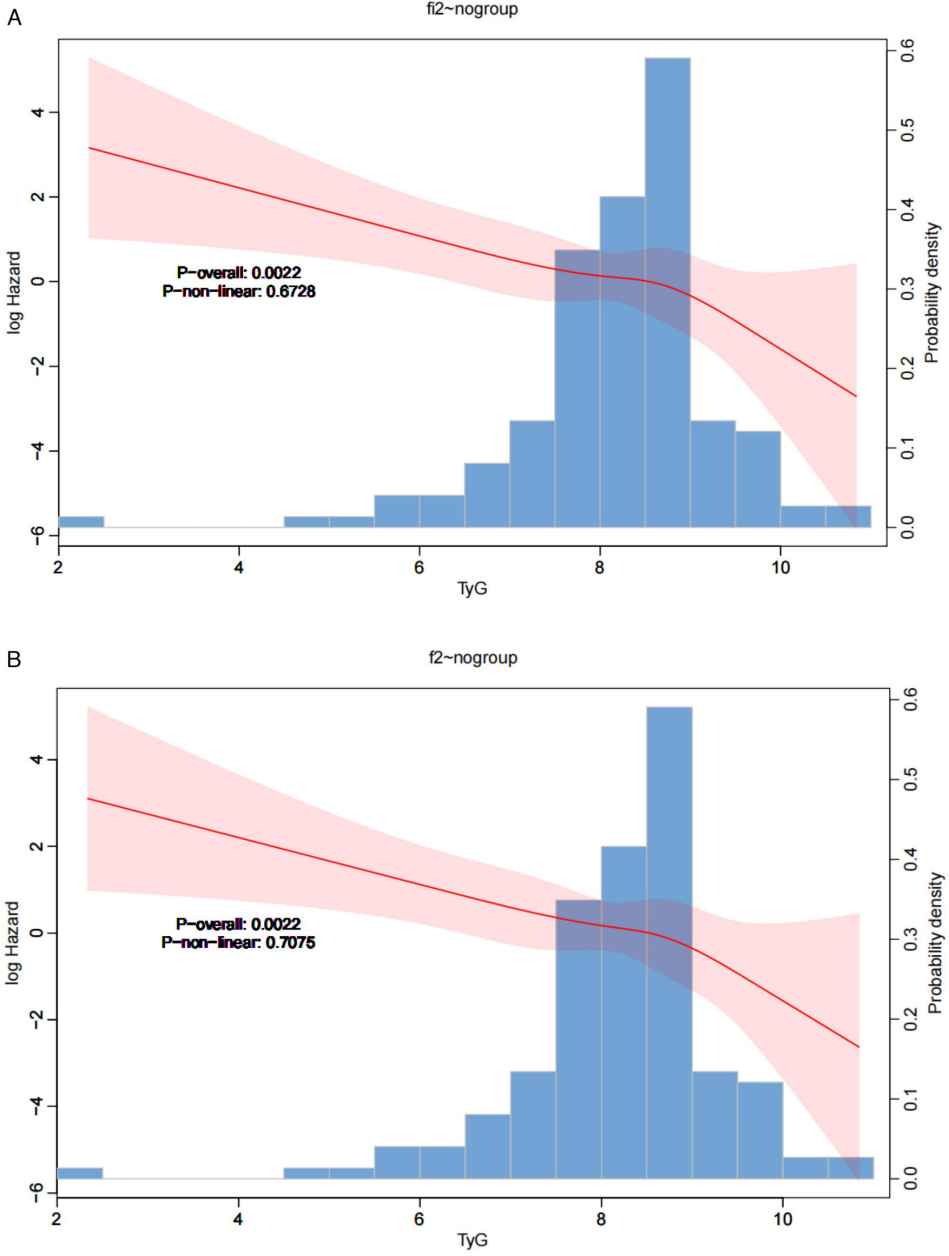
**(A)** Restricted cubic spline curve of the TyG index and the 30-day in-hospital mortality risk in pediatric sepsis patients. **(B)** Restricted cubic spline (RCS) curve of the TyG index and the 30-day ICU mortality risk in pediatric sepsis patients.

Similarly, the restricted cubic curve plots (Figures 2A,B) illustrate the correlation between the TyG index and the 30-day hospital mortality rate and the 30-day ICU mortality rate. The trend shown in this graph indicates that higher TyG index values are associated with a lower mortality rate in the ICU, which further validates the rationality of the TyG index as a relevant prognostic indicator.

### 3.3 Subgroup analysis of TyG index and 30-day mortality

**Figure 3:**
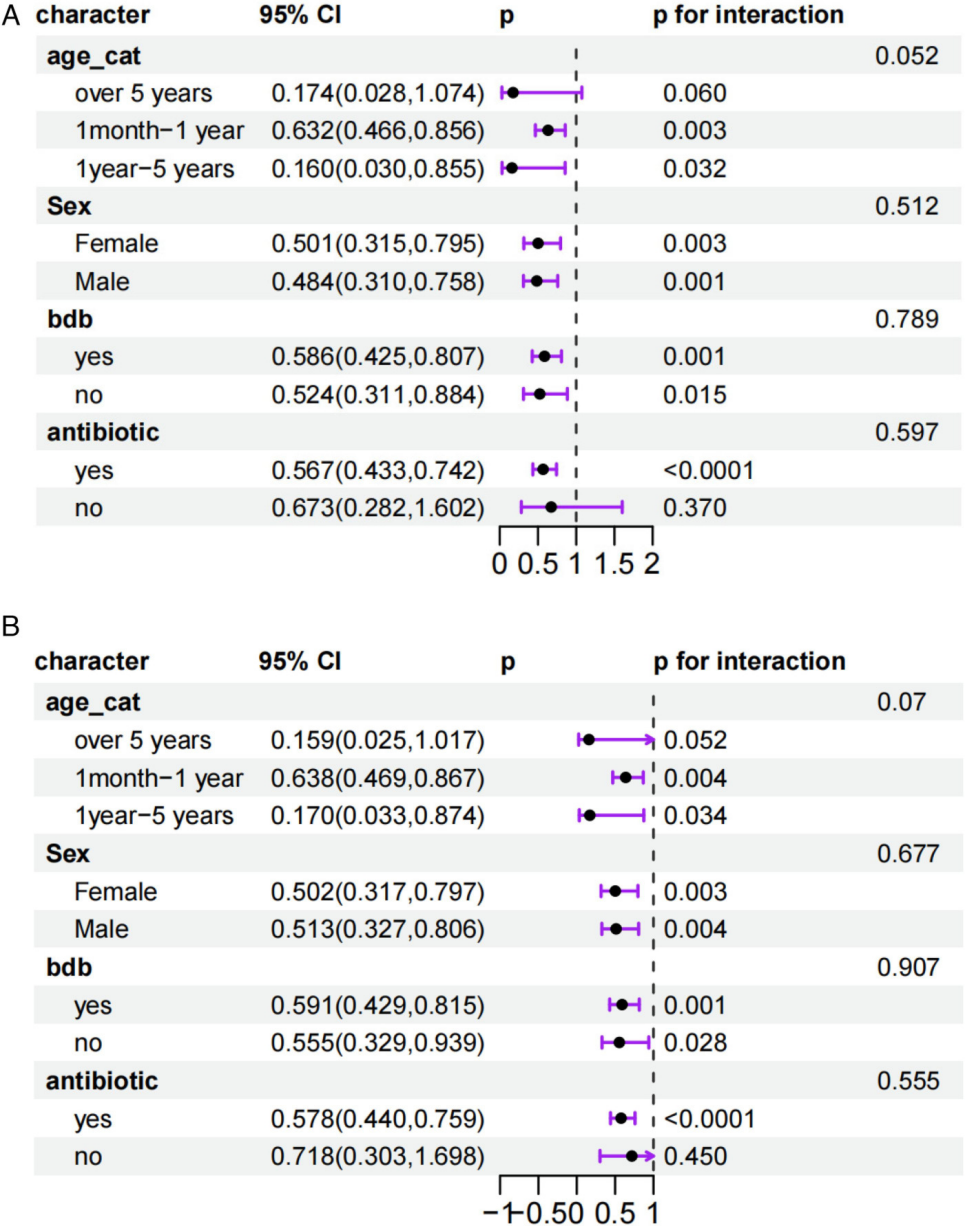
**(A)** Subgroup analysis of the relationship between the TyG index and 30-day in-hospital mortality in pediatric sepsis patients(bdb means albumin use). **(B)** Subgroup analysis of the relationship between the TyG index and 30-day ICU mortality in pediatric sepsis patients(bdb means Albumin use).

Figure 3A presents the subgroup analysis of the association between the TyG index and 30-day in-hospital mortality in pediatric sepsis patients. Subgroups were defined by age stratum, sex, albumin use and antibiotic use. In children >5 years, the hazard ratio (HR) was 0.174 (95% CI 0.028–1.074, *P* = 0.060), indicating a trend toward lower mortality that did not reach statistical significance. Among infants aged 1 month to 1 year, the HR was 0.632 (95% CI 0.466–0.856, *P* = 0.003), showing a significant reduction in mortality risk. Similarly, children aged 1–5 years had an HR of 0.160 (95% CI 0.030–0.855, *P* = 0.032), also denoting a significant decrease. When stratified by sex, girls exhibited an HR of 0.501 (95% CI 0.315–0.795, *P* = 0.003) and boys an HR of 0.484 (95% CI 0.310–0.758, *P* = 0.001), both indicating significantly lower mortality. Patients who received albumin had an HR of 0.586 (95% CI 0.425–0.807, *P* = 0.001), whereas those without albumin had an HR of 0.524 (95% CI 0.311–0.884, *P* = 0.015), both groups thus benefited. Among children given antibiotics, the HR was 0.567 (95% CI 0.433–0.742, *P* < 0.0001), reflecting a significant reduction, whereas no significant difference was observed in those without antibiotic therapy (HR 0.673, 95% CI 0.282–1.602, *P* = 0.370).

Figure 3B further presents the results of subgroup analysis on the relationship between TyG index and 30-day mortality in ICU among pediatric sepsis patients. For patients older than 5 years, the hazard ratio (HR) was 0.159 (95% CI: 0.025, 1.017) with a *P*-value of 0.052, suggesting a trend toward reduced mortality risk, though the difference did not reach statistical significance. For children aged 1 month to 1 year, the HR was 0.638 (95% CI: 0.469, 0.867) with a *P*-value of 0.004, indicating a significant reduction in mortality risk in this age group. For children aged 1 to 5 years, the HR was 0.170 (95% CI: 0.033, 0.874) with a *P*-value of 0.034, also showing a significant decrease in mortality risk. In female patients, the HR was 0.502 (95% CI: 0.317, 0.797) with a *P*-value of 0.004, indicating that female patients had a significantly lower mortality risk compared to male patients. For male patients, the HR was 0.513 (95% CI: 0.327, 0.806) with a *P*-value of 0.004, also showing a significant reduction in mortality risk. Patients who received albumin therapy had an HR of 0.591 (95% CI: 0.429, 0.815) with a *P*-value of 0.001, indicating a significant reduction in mortality risk. For patients who did not receive albumin therapy, the HR was 0.555 (95% CI: 0.329, 0.939) with a *P*-value of 0.028, also indicating a reduction in mortality risk. Patients who received antibiotic treatment had an HR of 0.578 (95% CI: 0.440, 0.759) with a *P*-value < 0.0001, indicating a significant reduction in mortality risk. For children who did not receive antibiotic treatment, the HR was 0.718 (95% CI: 0.303, 1.698) with a *P*-value of 0.450, indicating no significant difference in mortality risk.

From the above subgroup analysis, we can find that there is a certain association between TyG index and 30-day mortality in pediatric sepsis patients. The association between TyG index and mortality varies across subgroups stratified by age, gender, albumin use, and antibiotic use. Specifically, younger patients, female patients, those who used albumin, and those who used antibiotics had a relatively lower mortality risk. These findings suggest that TyG index may serve as a potential prognostic marker, however, its clinical application should take other patient characteristics into comprehensive consideration.

### 3.4 30-day ICU and in-hospital survival curves

**Figure 4:**
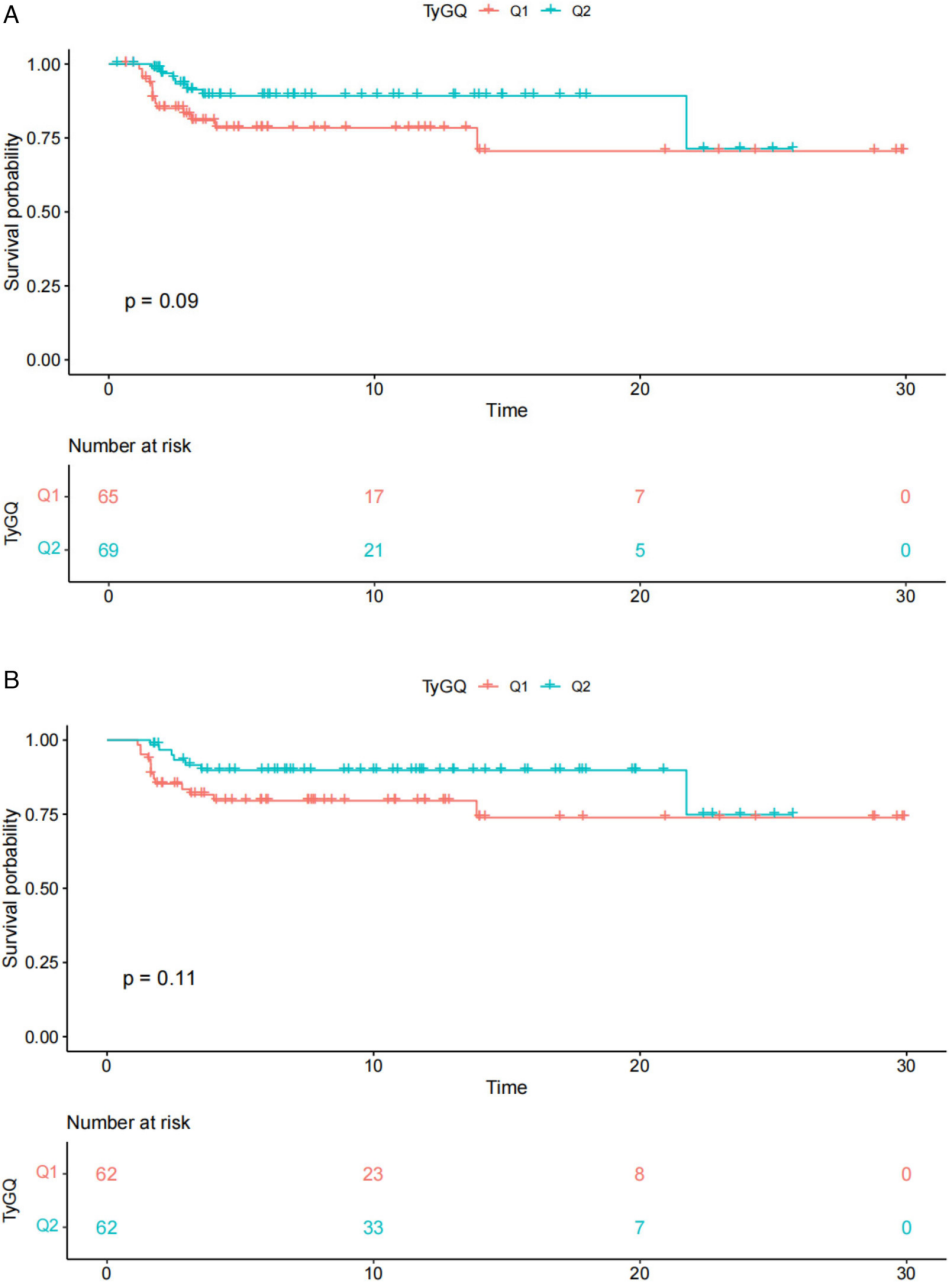
**(A)** Thirty-day ICU survival curve of pediatric sepsis patients. **(B)** Thirty-day in-hospital survival curve of pediatric sepsis patients.

To further evaluate the prognostic value of the triglyceride-glucose (TyG) index in pediatric sepsis, we used the Kaplan–Meier method to draw the 30-day survival curve and compared the survival probabilities among different TyG groups. We divided the children into Q1 (low TyG) group and Q2 (high TyG) groups based on the median of TyG, and conducted a 30-day follow-up for them. The endpoints for each group were death in the intensive care unit or death during the entire hospital stay. Figures 4A,B respectively show the 30-day survival curves in the ICU and throughout the hospital stay. Although the survival rate of the Q2 group (high TyG) was numerically higher than that of the Q1 group (low TyG), according to the Kaplan–Meier survival analysis, the difference between the two groups was not statistically significant (*P* > 0.05). Unfortunately, although a trend of higher TyG index being associated with better survival rate was observed, this association did not reach statistical significance in the sample of this study.

## 4 Discussion

This study found that in children with sepsis, an elevated triglyceride-glucose (TyG) index was associated with a higher risk of 30-day mortality, and this association remained consistent across different age groups, genders, and treatment subgroups. This finding stands in stark contrast to the conclusion from adult studies that “the higher the TyG index, the poorer the prognosis.” Recent studies have shown that each 1-unit increase in the TyG index is associated with an 18%-22% increase in the risk of ICU mortality (9, 10). However, the pathophysiology of pediatric sepsis is characterized by “a surge in metabolic demand and rapid depletion of hepatic glycogen and fat stores" (19). A high TyG index may reflect that the children still have sufficient metabolic substrates, thus exhibiting a protective effect. This age-dependent difference suggests that the prognostic significance of the TyG index needs to be verified separately in different populations and cannot be directly extrapolated from adult data.

Based on a comprehensive consideration of existing literature and biological plausibility, Model 2 further adjusted for additional laboratory indicators on the basis of Model 1. This adjustment aimed to control for these known pathophysiological confounders closely associated with sepsis prognosis. The TyG index remained independently and significantly associated with the outcome after this adjustment, indicating that its prognostic value is largely independent of traditional markers of inflammatory severity and organ dysfunction. This finding reinforces the notion that the TyG index may represent a unique dimension in sepsis, rather than merely serving as another surrogate for disease severity. It suggests that relatively higher TyG index levels may play a protective role during acute stress in pediatric sepsis patients, contrasting with its observed role as a marker of insulin resistance and poor prognosis in adults with chronic diseases (20). Future studies should focus on elucidating the specific biological mechanisms underlying this protective association.

In critically ill adult patients, the TyG index is typically regarded as a marker of insulin resistance and metabolic disorders. Its elevation is usually associated with chronic inflammation, cardiovascular diseases, and an increased risk of all-cause mortality. However, the results of this study indicate that in the pediatric sepsis population, a higher TyG index shows a protective effect, which may be closely related to its metabolic regulatory function under acute stress. The emerging multi-omics research has further revealed the molecular basis of age-related metabolic heterogeneity in sepsis. Comprehensive multi-omics analysis has identified different metabolic and immune characteristics of sepsis molecular subtypes, which may have certain reference significance for explaining the differences in TyG index preconditions shown by different age groups (21–23). Such studies emphasize that metabolic dysregulation in sepsis is not a uniform process but a subtype-specific phenomenon. For instance, Ning et al. used comprehensive multi-omics integration and single-cell transcriptome analysis to prove that sepsis patients exhibit subtype-specific differences, with the marker gene LILRA5 being closely related to sepsis (21). Other studies further confirmed that the comprehensive immune landscape of sepsis in different age groups has significant differences, and these findings may provide guidance for patient stratification and clinical applicable detection methods for prognosis, and offer a framework for precise immune regulation strategies in sepsis care (24). However, the underlying biological mechanism still requires further research and direct evidence to support it. We look forward to future studies that can further reveal the biological basis of the protective effect of the TyG index in pediatric sepsis and provide new insights and intervention strategies for clinical practice.

Although a trend of higher TyG index being associated with better survival rates was observed in Figure 4, this association did not reach statistical significance in the sample of this study. This finding suggests that although the TyG index may be related to the short-term survival rate of children with sepsis, this relationship may be influenced by other unmeasured factors, or a larger sample size is needed to confirm it. Therefore, although the TyG index shows the potential to be positively correlated with survival rates, in the current research context, we cannot assert that it is an independent prognostic indicator. Future research may need to further explore the interaction between the TyG index and other potential prognostic factors, as well as verify its prognostic value in larger or more diverse populations. Survival curves (Figures 4A,B) provide us with an initial insight into the potential prognostic role of the TyG index and emphasize the importance of considering metabolic factors in the management of pediatric sepsis. However, they also remind us that more evidence is needed to support the effectiveness of the TyG index as a clinical prognostic tool before it can be used as such. Furthermore, although this study validated the prognostic value of the TyG index in multiple models, several limitations exist. First, this study was retrospective, potentially introducing selection bias and information bias. Second, the sample size was relatively small, and data were derived from a single database, which may limit the generalizability of the results. Additionally, although we adjusted for multiple confounding factors in the models, other unaccounted-for factors may still influence the results. Finally, this study did not delve into the potential biological mechanisms underlying the association between the TyG index and sepsis prognosis, which warrants further exploration in future research.

Given the findings of this study and the current state of research in this field, future studies can be conducted in the following key directions to further elucidate the prognostic value of the triglyceride-glucose (TyG) index in pediatric sepsis patients and its potential mechanisms. First, mechanistic studies are central to understanding the relationship between the TyG index and sepsis prognosis. While previous studies have shown that insulin resistance and dyslipidemia are closely associated with the development of various chronic diseases, their specific mechanisms in sepsis remain unclear. Future studies should delve into the biological mechanisms underlying the association between the TyG index and sepsis prognosis, including the roles of insulin resistance and dyslipidemia in sepsis. This may involve research across multiple levels, such as inflammatory responses, oxidative stress, cellular signaling pathways, and metabolomics. By elucidating these mechanisms, more precise targets for clinical intervention can be identified. Second, prospective studies are a critical step in validating the prognostic value of the TyG index. Although this study preliminarily validated the association between the TyG index and 30-day mortality in pediatric sepsis patients through a retrospective cohort study, the inherent limitations of retrospective studies (such as selection bias and information bias) may affect the accuracy and generalizability of the results. Therefore, prospective cohort studies should be conducted in the future, involving larger sample sizes and more diverse populations, to validate the prognostic value of the TyG index in different clinical settings and patient groups. This will help further confirm the reliability of the TyG index as an independent prognostic marker and provide stronger evidence for clinical practice. Finally, intervention studies are a crucial component in applying the TyG index to clinical practice. Current research has primarily focused on the predictive value of the TyG index, but its potential for clinical intervention has not been fully explored. Future studies should assess whether interventions targeting TyG index-related metabolic abnormalities can improve the prognosis of sepsis patients. For example, can improving insulin resistance or regulating lipid levels reduce mortality and complication rates in sepsis patients? Such studies not only help validate the clinical application value of the TyG index but may also provide new strategies for the treatment of sepsis. In conclusion, future research should be conducted from the perspectives of mechanism, validation, and intervention, in order to comprehensively evaluate the prognostic value of the TyG index in pediatric sepsis patients and its potential clinical application prospects. This will help advance research on sepsis prognostic markers and provide new insights and methods for improving clinical outcomes in sepsis patients.

## 5 Conclusions

This study found that in pediatric sepsis patients, a higher TyG index was associated with a lower 30-day mortality rate, and this association remained consistent across different age groups, genders, and treatment subgroups. This result differs from the conclusion in adult studies that TyG index is associated with adverse prognosis. This suggests that the relationship between TyG index and the prognosis of pediatric sepsis may be influenced by other unmeasured factors, or a larger sample size is needed to confirm it.

## Data Availability

Publicly available datasets were analyzed in this study. This data can be found here: http://pic.nbscn.org/.
